# Menstrual cycle variations in stress vulnerability and sociability relate to mental health symptoms and libido

**DOI:** 10.1038/s44294-026-00140-z

**Published:** 2026-04-17

**Authors:** Belinda Pletzer, Tobias Hausinger, Neele Thoms, Cosima Gierg, Adriene M. Beltz

**Affiliations:** 1 Department of Psychology, University of Salzburg, Salzburg, Austria; 2 Centre for Cognitive Neuroscience, University of Salzburg, Salzburg, Austria; 3 Department of Psychology, University of Michigan, Ann Arbor, MI USA

**Keywords:** Reproductive biology, Reproductive disorders

## Abstract

Previous studies have suggested the high-estradiol peri-ovulatory phase as a window of resilience, and the luteal phase, characterised by changing progesterone levels, as a window of vulnerability to stress-related disorders. Using an intensive longitudinal design over 75 days, the current study explored whether stress vulnerability and resilience were reflected in menstrual cycle-related shifts in personality facets. In a sample of 68 healthy female participants aged 18 to 35 years, we observed increased stress vulnerability, decreased sociability, and decreased non-antagonistic orientation during the peri-menstrual phase. Changes in stress vulnerability were both preceded and followed by changes in mental health symptoms (depression, anxiety, mood lability, irritability), while changes in mental health symptoms followed—but did not precede—sociability and non-antagonistic orientation. These results suggest that personality facet shifts along the menstrual cycle precede changes in mental health symptoms, though the personality facets explored in the current study did not fully account for menstrual cycle-related changes in these symptoms.

## Introduction

The menstrual cycle is an excellent model for studying the neuroendocrine modulation of affective and cognitive processes, given that it presents with distinct peaks for estradiol and progesterone during different phases. While both hormones are at their lowest at the beginning of the cycle, estradiol production by the dominant follicle increases during the late follicular phase, peaking shortly before ovulation, while progesterone production by the corpus luteum starts after ovulation, peaking about a week after ovulation in the mid-luteal phase. This mid-luteal progesterone peak is accompanied by heightened estradiol levels, and both hormones drop in the premenstrual phase. These ovarian hormone fluctuations have been consistently linked to neuroplastic changes in various brain circuits in both animal studies of the estrous cycle^1,2^ and human studies of the menstrual cycle (see Pletzer et al.^3^ for a review). Indeed, several recent large-scale behavioural studies with innovative designs have illuminated the emotional and behavioural changes associated with windows of increased neuroplasticity surrounding hormonal peaks and linked them to vulnerability or resilience to stress-related disorders^3–5^.

Among these studies, those with intensive longitudinal designs (with many densely spaced repeated within-person assessments) capturing daily hormone-related variability in human behaviour have proven particularly informative, as they can track person-specific rises and falls in hormone levels and behaviour^6,7^. Results from studies with intensive longitudinal designs have revealed increased negative mood during the pre-menstrual and/or menstrual phase compared to other phases^8–10^, though some studies do not observe negative affect fluctuations along the menstrual cycle^11^. Intensive longitudinal studies have also revealed increased positive affect^12^ and libido during the peri-ovulatory phase compared to other phases^13–15^, but little to no changes in cognitive functions across the cycle^3^. Even though this latter result is debated in the literature^16^, methodological variations across studies may explain the discrepancies in their findings, and recent meta-analyses provide converging evidence for small-to-null results across participants^17,18^. However, these studies did not operationalize stress, and the neuroplasticity literature suggests that changing neuroendocrine milieus along the menstrual cycle affect how stressful experiences are processed; consequently, stressful experiences may exacerbate menstrual cycle-related changes in affective and cognitive processes.

Thus, findings from intensive longitudinal studies are consistent with other work indicating that the mid-to-late luteal phase may be a window of vulnerability for the emergence of symptoms typical for stress-related disorders^10,19^. During the mid-luteal phase, increased salience for negative emotional stimuli^20^ (see Hsu et al.^21^ for a meta-analysis) and increased self-referential processing^22^ have been observed. This may result in increased vulnerability to develop affective symptoms^10^ that manifest most prominently in the late luteal phase, i.e., before menstruation. The neuroendocrinological mechanisms relating to this increased stress vulnerability are not fully understood^23^. However, the severity of premenstrual mental health symptoms—including depression, anxiety, mood, and irritability—varies across women, with an estimated 4–8% fulfilling the criteria for a clinical diagnosis of premenstrual dysphoric disorder (PMDD)^24^. Still, sub-clinical forms with fewer or less severe symptoms may impact quality of life and are thus summarised as premenstrual syndrome (PMS). Importantly, changes in mental health symptoms, affect, and mood in the sub-population experiencing PMS/PMDD can be strong enough to drive group-level differences even though most women in a sample do not experience emotional changes along the menstrual cycle^25^. The fact that PMS/PMDD is largely underdiagnosed^26^ may explain the discrepancies regarding negative affect changes during the premenstrual phase between studies^9–11^. Thus, when evaluating stress vulnerability in the premenstrual phase, it is important to take these inter-individual variations in symptom trajectories into account.

Vice versa, the peri-ovulatory phase is viewed as a window of resilience towards stress-related disorders owing to its neuroplastic opportunities for adaptation^3,5,27^. Much of this neuroplastic potential is attributed to the increased estradiol production by the dominant follicle. Beyond the neuroplastic effects of estradiol, many of its associations with mood and sexual interest have been related to its stimulation of kisspeptinergic neurons related to ovulation. Increased kisspeptin signalling has been identified as the mechanism underlying changes in sexual behaviour around ovulation and is also associated with reduced depression and anxiety^28^. Thus, ovulation is now considered an important sign of female health^29^.

Taken together, findings from intensive longitudinal studies, neuroscience, and neuroendocrinology make apparent that stress vulnerability or resilience fluctuates, or is state-like, along the menstrual cycle. However, the psychological literature largely portrays resilience as an inter-individual trait^30^. Extensive literature outlines the aspects of personality that are related to psychological resilience on a trait-level (see Oshio et al.^31^ for a meta-analysis). Neuroticism, for instance, reflects stress vulnerability, whereas extraversion, openness, agreeableness, and conscientiousness appear to foster psychological resilience. However, the stability of resilience over time seems to be a matter of debate and may be the deciding factor for its conceptualization either as a personality trait or as a learned skill that can evolve with experience^30^. This differentiation stems from a traditional view on personality, which in its original conceptualization is considered stable (for notable exceptions, see Roberts et al.^32^). Consequently, no previous study has investigated personality shifts with respect to the menstrual cycle. If anything, personality traits like neuroticism have been used to mark predispositions to behavioural changes along the menstrual cycle^33^. However, by drawing on the concepts of (stress) vulnerability and resilience and framing them as variable over time, menstrual cycle research intersects with important recent developments in personality research.

The dynamic aspects of personality have recently become a subject of investigation^34^, and research on personality states is accumulating, evidencing substantial variability in day-to-day manifestations of various personality facets^34–37^. This presents measurement challenges, given that the global five-factor structure does not replicate at the intra-individual level^38–41^. However, we recently showed that certain lower order aspects of personality (i.e., personality facets) reliably vary across time-points and individuals and can thus be meaningfully assessed using a sub-scale derived from a traditional trait questionnaire^42^. Specifically, the neuroticism-related facet stress vulnerability^42^ can be meaningfully captured by the same items at both the trait and state level. This is particularly relevant in the context of stress vulnerability and resilience, as it has been unclear whether these facets are causes or consequences of individual differences in environmental challenges^30^. Indeed, personality states have been related to several person-specific and environmental factors, like daily affect, stressors, and emotion regulation^43,44^. For example, Wright et al.^45^ estimate that 40-50% of changes in inter-personal behaviour are due to daily shifts in personality related to stress. Yet, it is still unclear whether daily personality shifts are associated with neuroendocrine modulations along the menstrual cycle. This is important to clarify because a deeper understanding of the associations between hormonal fluctuations and personality states may, in the long term, provide valuable insights for the prevention or treatment of stress-related disorders, thus fostering mental health and well-being.

In the present study, we explored menstrual cycle-related variations in personality facets relevant for psychological resilience and stress vulnerability in an intensive longitudinal study while accounting for individual trajectories. Specifically, we employed a personality scale previously optimised for intensive longitudinal assessment^42^ in a sample of 68 women over 75 days (daily), thus capturing two to three menstrual cycles. The scale encompasses the neuroticism-related facet stress vulnerability, the extraversion-related facet sociability, the agreeableness-related facet non-antagonistic orientation, as well as the conscientiousness-related facets dependability and achievement. In addition, we tracked positive and negative affect, mental health symptoms typically associated with the premenstrual phase, and libido, given their established changes across the menstrual cycle.

We hypothesised that stress vulnerability increases during the mid-to-late luteal phase, while personality facets related to increased resilience, such as sociability, non-antagonistic orientation, dependability, and achievement orientation, increase during the peri-ovulatory phase. We also explored whether menstrual cycle-related changes in stress vulnerability relate to mental health symptoms and negative affect, and whether menstrual cycle-related changes in personality facets regarding resilience relate to libido and positive affect. Specifically, we performed time-lagged analyses to address whether changes in personality facets precede or follow changes in mental health symptoms, libido, and affect.

## Results

### Menstrual cycle comparisons

Descriptive statistics of dependent variables per cycle phase are summarised in Table 1.

**Table 1:** Descriptive statistics of study variables by cycle phase

	Mid-follicular [days + 4 to −20]	Peri-ovulatory [days −15 to −12]	Mid-luteal [days −9 to −5]	Peri-menstrual [days −3 to + 2]
Estradiol [pg/ml]	0.95 ± 0.57	1.23 ± 0.69	1.16 ± 0.74	0.98 ± 0.55
Progesterone [pg/ml]	34.06 ± 52.74	63.73 ± 84.97	127.14 ± 104.74	51.85 ± 54.89
Stress Vulnerability [1–5]	2.03 ± 0.79	1.94 ± 0.74	1.98 ± 0.81	2.06 ± 0.82
Sociability [1–5]	3.35 ± 0.56	3.35 ± 0.57	3.36 ± 0.61	3.24 ± 0.63
Nonantagonistic O. [1–5]	4.12 ± 0.67	4.11 ± 0.71	4.07 ± 0.74	4.04 ± 0.71
Achievement O. [1–5]	3.62 ± 0.66	3.58 ± 0.66	3.56 ± 0.65	3.55 ± 0.62
Dependability [1–5]	3.83 ± 0.58	3.80 ± 0.56	3.77 ± 0.60	3.76 ± 0.53
Mental Health S. [1–6]	1.85 ± 0.65	1.78 ± 0.63	1.80 ± 0.71	1.93 ± 0.70
Physical Symptoms [1–6]	1.43 ± 0.45	1.38 ± 0.45	1.39 ± 0.43	1.60 ± 0.54
Positive Affect [1–5]	2.81 ± 0.71	2.80 ± 0.74	2.79 ± 0.76	2.76 ± 0.71
Negative Affect [1–5]	1.47 ± 0.36	1.46 ± 0.38	1.48 ± 0.46	1.51 ± 0.42
Libido [1–5]	2.25 ± 0.83	2.42 ± 0.82	2.11 ± 0.73	1.95 ± 0.69

Values represent means ± standard deviations.

*O* Orientation, *S* Symptoms.

To address menstrual cycle-related changes in these variables while accounting for individual trajectories, we used linear mixed effects models including random slopes and Bayesian analyses. Statistical parameters of these comparisons are summarised in Table 2.

**Table 2:** Results of study variable comparisons across menstrual cycle phases

	Cycle phase fixed effect			Cycle phase random effect		Trait-slope^a^ correlation
	**η**_**p**_**²**	***F***	**BF**_**10**_	**L.ratio**	**BF**_**10**_	***r***
Estradiol	0.02	18.40^***^	5*10^**8**^ ± **1.53%**	44.05^***^	**0.46** ± **1.64%**	0.26
Progesterone	0.05	37.73^***^	1*10^**26**^ ± **2.18%**	437.15^***^	2*10^**65**^ ± **2.07%**	0.62^***^
Stress vulnerability	<0.01	3.43^*^	**0.50** ± **2.45%**	120.48^***^	9*10^**22**^ ± **1.81%**	−0.28
Sociability	0.01	4.72^**^	**5.88** ± **1.92%**	160.32^***^	3*10^**32**^ ± **1.68%**	−0.19
Nonantagonistic O.	0.01	3.96^*^	**0.76** ± **1.59%**	96.24^***^	3*10^**16**^ ± **1.70%**	0.03
Achievement O.	<0.01	0.59	0.02 ± 1.51%	194.27^***^	4*10^38^ ± 2.10%	0.18
Dependability	<0.01	0.40	0.01 ± 1.75%	250.96^***^	2*10^49^ ± 1.98%	0.01
Mental Health S.	0.01	8.82^***^	6*10^**2**^ ± **1.80%**	126.28^***^	5*10^**19**^ ± **2.05%**	−0.29
Physical symptoms	0.03	24.26^***^	6*10^**14**^ ± **1.41%**	295.23^***^	4*10^**55**^ ± **1.61%**	−0.32
Positive affect	<0.01	1.70	0.04 ± 1.24%	240.08^***^	2*10^48^ ± 1.25%	0.09
Negative affect	<0.01	3.19^*^	0.09 ± 1.14%	201.29^***^	2*10^36^ ± 1.50%	−0.17
Libido	0.03	14.32^***^	1*10^**6**^ ± **1.49%**	97.37^***^	9*10^**17**^ ± **1.66%**	0.28

^a^Traits were calculated as mean values over all days, slopes were calculated as strongest difference between phases, i.e., as difference between peri-menstrual and peri-ovulatory values, with the exception of progesterone, for which the strongest increase was observed in the mid-luteal phase.

*O* Orientation, *S* Symptoms, *BF*_10_ Bayes factor in support of the alternative hypothesis, *Lratio* likelihood ratio. ^*^p_FDR_ < 0.05, ^**^p_FDR_ < 0.01, ^***^p_FDR_ < 0.001.

**Fig. 1:**
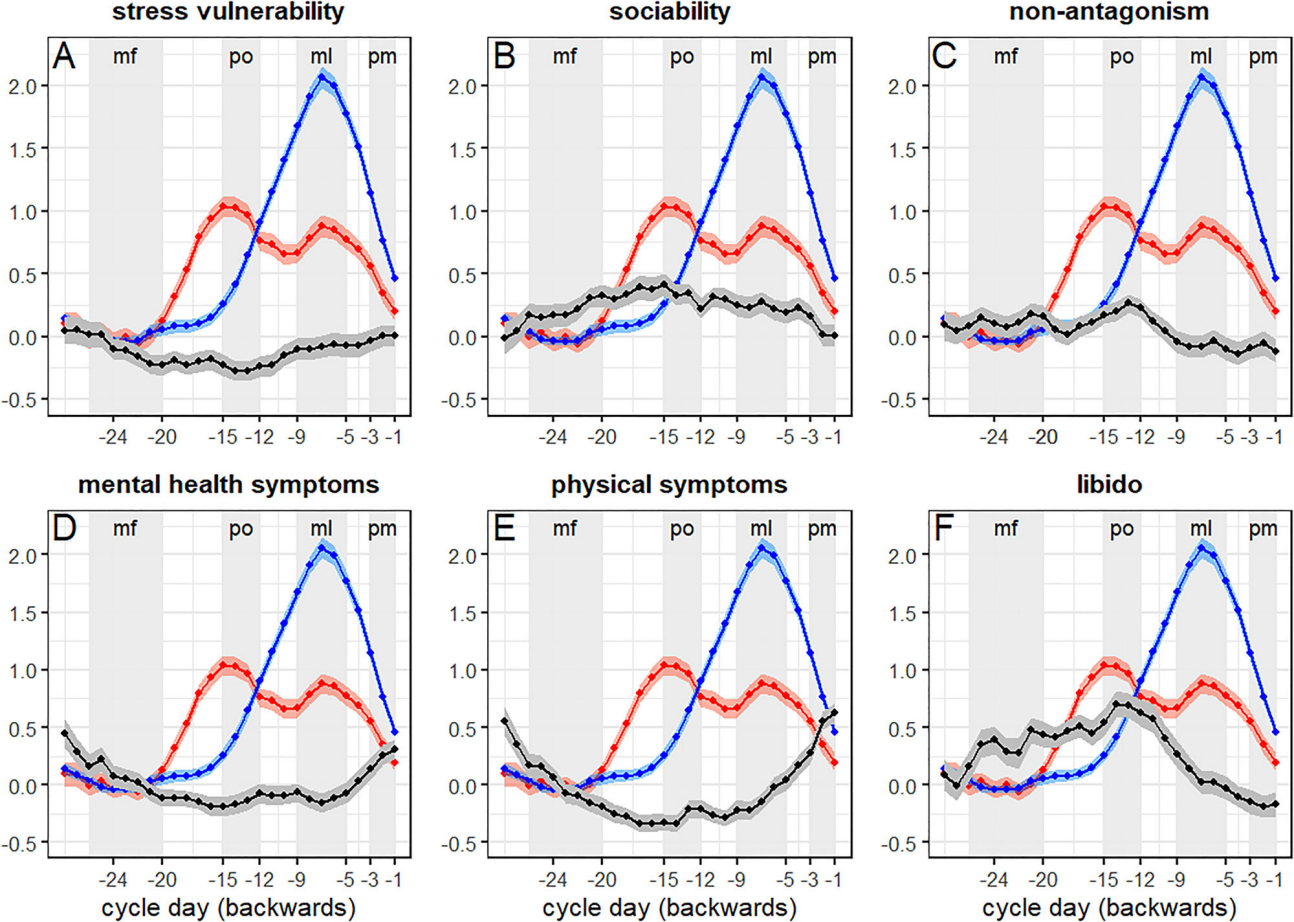
Average trajectories of personality expressions (upper panel) and health symptoms (lower panel) along the menstrual cycle (grey). paralleled by average *estradiol* (red) and *progesterone* (blue) fluctuations. **A** stress vulnerability, **B** sociability, C: non-antagonism, **D** mental health symptoms, **E** physical symptoms, **F** libido. The x-axis shows days to next menstruation. Given comparable luteal phase length between subjects, longer cycles were cut at −28 days for illustration purposes. Variables were smoothed by a five-point moving average and individually scaled for display purposes. Grey areas mark the selected time windows for menstrual cycle phases according to the definition of Schmalenberger et al (2021). Mf mid-follicular, po peri-ovulatory, ml mid-luteal, pm peri-menstrual. Please note that the mid-follicular window depends on the individual cycle length and the peri-menstrual window extends to the first two days of menses in the next cycle.

Estradiol and progesterone followed the expected patterns over the menstrual cycle (see Table 2; compare Table 1 and Fig. 1A–D) with significant increases in estradiol during the peri-ovulatory and mid-luteal phases compared to mid-follicular and peri-menstrual phases (all |*b*| > 0.05, all |*z*| > 3.60, all *p*_*Tukey*_ < 0.002), and a significant increase in progesterone during the mid-luteal phase compared to the other cycle phases (all |*b*| > 0.10, all |*z*| > 7.63, all *p*_*Tukey*_ < 0.001). Estradiol levels did not differ significantly between mid-luteal and peri-ovulatory phases (*b* = −0.12, *z* = −1.68, *p*_*Tukey*_ = 0.33).

In testing hypotheses (see Tables 1 and 2), fixed effects analyses revealed significant menstrual cycle-related changes for *s*tress vulnerability (Fig. 1A) and sociability (Fig. 1B), as well as non-antagonistic orientation, but not achievement orientation or dependability. As expected, pairwise comparisons revealed higher average stress vulnerability scores in the peri-menstrual phase compared to the peri-ovulatory phase (*b* = 0.15, *z* = 3.04, *p*_*Tukey*_ = 0.01), and reduced average sociability and non-antagonistic orientation during the peri-menstrual phase compared to the mid-follicular (both *b* = −0.09, both *z* = −2.60, both *p*_*Tukey*_ < 0.05) and peri-ovulatory phases (both *b* < −0.12, both *z* < −3.13, both *p*_*Tukey*_ < 0.01). Details on associations with estradiol and progesterone are reported in Supplementary Table 1. Interestingly, stress vulnerability showed no association to sex hormones, while both sociability and non-antagonistic orientation were lower with higher progesterone levels.

In addition, mental health and physical symptoms typically associated with the premenstrual phase, libido, and negative affect—but not positive affect—showed significant changes along the menstrual cycle. As expected, pairwise comparisons confirmed that mental health and physical symptoms were significantly increased in the peri-menstrual phase compared to the other cycle phases (all *b* > 0.10, all *z* > 5.26, all *p* < 0.001; Fig. 1C), while libido was significantly increased during the peri-ovulatory phase compared to the mid-luteal and peri-menstrual phase (all |*b*| > 0.35, all |*z*| > 3.75, all *p* < 0.001; Fig. 1D). Mental health and physical symptoms were negatively, and libido positively related to estradiol (compare Supplementary Table 1).

Except for stress vulnerability and non-antagonistic orientation, Bayesian analyses supported frequentist statistics. Bayes factors > 6 indicated that models including menstrual cycle-related changes are at least 6 times more likely than models without menstrual cycle-related changes for hormone levels, sociability, mental health symptoms, and libido. Bayes factors < 0.10 indicated that models without menstrual cycle-related changes were at least 10 times more likely than models including menstrual cycle-related changes for achievement orientation, dependability, positive and negative affect. The Bayes factors for stress vulnerability and non-antagonistic orientation were inconclusive and did not provide even anecdotal support for one model over the other.

**Fig. 2:**
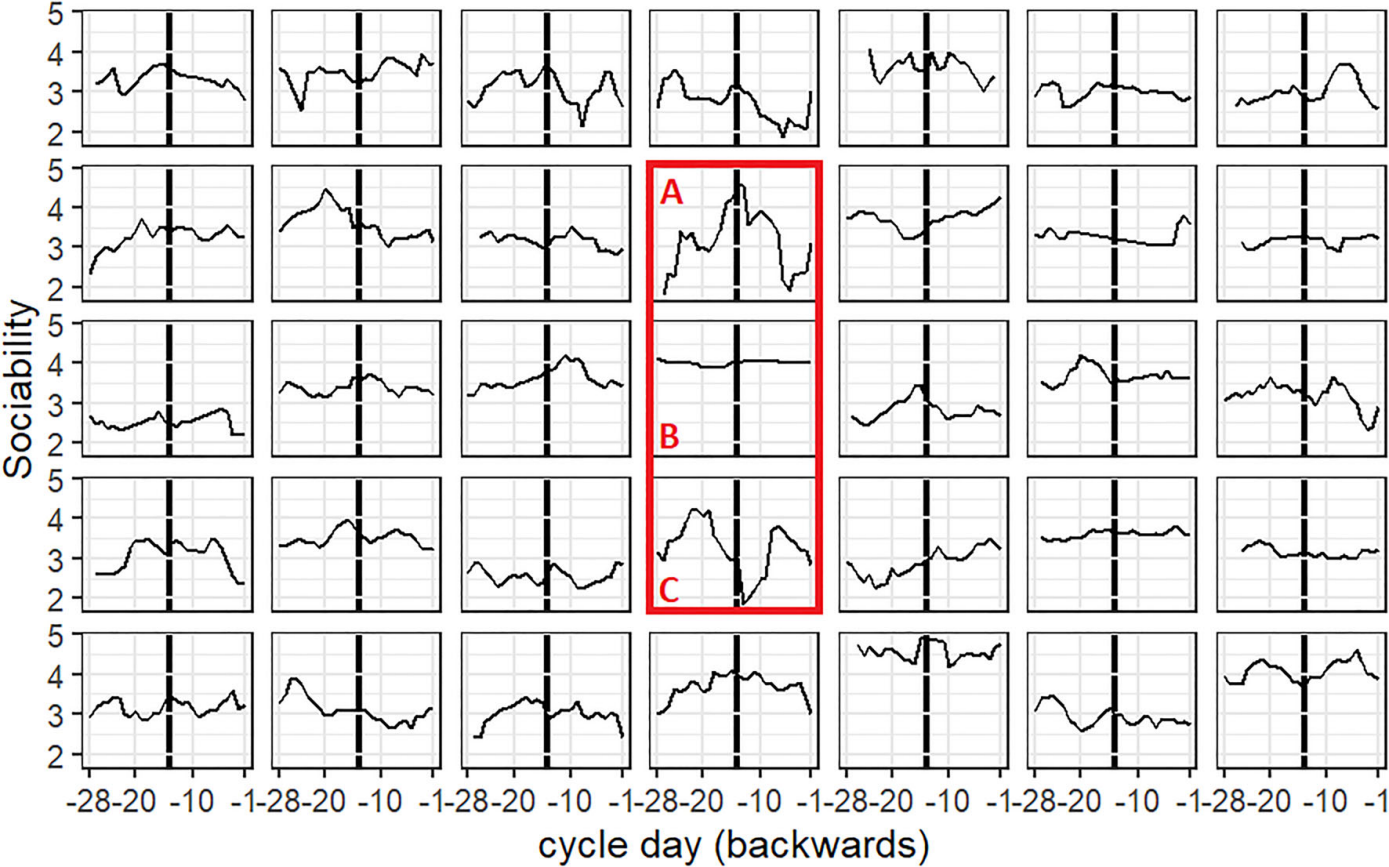
Individual variability in menstrual cycle-related fluctuations in sociability. Each plot represents one person. The red rectangle highlights three participants with different trajectories. The *x*-axis shows days to next menstruation, the *y*-axis sociability scores. Given comparable luteal phase length between subjects, longer cycles were cut at -28 days for illustration purposes. Ovulation is marked at day -14 as a thick vertical black line.

Random slopes indicated substantial variability in the relation between menstrual cycle-related changes and personality facet expressions across participants (Table 2). In Fig. 2, this variability was visualised for the trajectories of sociability in 35 representative participants. Most participants showed some sort of peak in sociability around ovulation, most clearly defined in participant A. However, some participants hardly showed any changes in sociability along the menstrual cycle (e.g., participant B), and a few participants even showed a drop in sociability around ovulation (e.g., participant C).

To explore whether this inter-individual variability in menstrual cycle trajectories was related to trait-values, trait-slope associations were calculated by correlating mean variable values across all days to their strongest change between cycle phases, *i.e*., the change from the peri-ovulatory to the peri-menstrual phase. Associations between intercept and slope were non-significant for all variables except for progesterone, for which a stronger mid-luteal peak was also reflected in stronger average values.

To further explore potential factors explaining this inter-individual variability in menstrual cycle trajectories, we performed exploratory moderation analyses by severity of premenstrual mental health symptoms. These analyses revealed that menstrual cycle-related changes in stress vulnerability, as well as positive and negative affect, were exacerbated in participants with stronger mental health symptoms (all *F* > 4.35, all *p*_*FDR*_ < 0.03), whereas menstrual cycle-related changes in other personality facets and libido were not significantly moderated by severity of mental health symptoms (for details see Supplementary Table 2).

### Relationship to ovarian hormones, mental health symptoms, and libido

To evaluate associations between intra-individual fluctuations in personality facets as well as mental health symptoms and libido, Table 3 lists zero-order correlations between those individually centred variables demonstrating significant associations to the menstrual cycle. Associations to ovarian hormones were negligible and are thus not evaluated further. Associations among personality facets were moderate. A strong intra-individual correlation was observed between mental health symptoms and stress vulnerability, whereas associations to sociability and non-antagonistic orientation were moderate. Associations of personality facets, as well as mental health symptoms and physical symptoms to libido were small.

**Table 3:** Correlation between individually centred menstrual cycle-related variables

	E	P	V	S	N	MHS	PHS
Progesterone (P)	0.25^***^						
Stress Vulnerability (V)	0.02	0.01					
Sociability (S)	−0.01	−0.01	−0.31^***^				
Nonantagonism (N)	−0.01	−0.04	−0.36^***^	0.21^***^			
Mental Health Symptoms (MHS)	−0.01	−0.01	0.55^***^	−0.34^***^	−0.27^***^		
Physical Symptoms (PHS)	−0.04	−0.04	0.15^***^	−0.09	−0.07		
Libido	0.05	−0.01	−0.10^***^	0.12^***^	0.04	−0.13^***^	−0.10^***^

^***^p_FDR_ < 0.001.

To evaluate whether fluctuations in personality facets were more strongly tied to the menstrual cycle or to mental health symptoms and libido, we hierarchically entered mental health symptoms and libido as additional predictors to the linear mixed effects models evaluating menstrual cycle-related changes in personality facets (Table 4). Results clearly demonstrated that mental health symptoms, and to a lesser extent libido, show much stronger associations to personality facets than to menstrual cycle phases. In fact, associations of personality facets to menstrual cycle phases were not significant when adjusting for mental health symptoms.

**Table 4:** Association of personality facets to cycle phases after adjusting for mental health symptoms and libido

	Stress vulnerability		Sociability		Nonantagonistic O.	
	**η**_**p**_**²**	***F***	**η**_**p**_**²**	***F***	**η**_**p**_**²**	***F***
Step 1:						
Cycle phase	< 0.01	3.43^*^	0.01	4.72^**^	0.01	3.96^**^
Step 2:						
Cycle Phase	0.01	0.28	0.01	1.90	0.01	1.80
Mental Health Symptoms	0.37	1380.67^***^	0.13	345.70^***^	0.08	231.95^***^
Step 3^a^:						
Cycle Phase	0.01	0.84	0.01	0.49	0.01	1.46
Mental Health Symptoms	0.44	1044.51^***^	0.15	207.34^***^	0.11	191.56^***^
Libido	0.01	7.06^**^	0.01	9.16^**^	< 0.01	5.91^*^

*p_FDR_ < 0.05, **p_FDR_ < 0.01, ***p_FDR_ < 0.001. *O* Orientation.

^a^This model was evaluated only in the subsample for which libido scores were available.

To address the directionality of the associations between personality and mental health symptoms, we performed time-lagged analyses. Specifically, we were interested whether daily personality facet expressions were predicted by mental health symptoms from the previous day or vice versa. Given that all variables strongly predicted their own next day value (all *b* > 0.19, all *t* > 9.39, all *p* < 0.001), these models were adjusted by same day values of the predictors. Thus, they evaluated whether previous day mental health explained variability in personality beyond the variance explained by same day mental health and vice versa. Results are summarised in Table 5. Stress vulnerability was the only personality facet related to previous-day mental health symptoms, but it also predicted next-day mental health symptoms, suggesting that associations between stress vulnerability and mental health problems are circular. Sociability was not related to previous day mental health symptoms, but it did predict next day mental health symptoms, suggesting reduced sociability preceded mental health problems, but not vice versa. Finally, non-antagonistic orientation was only related to mental health symptoms on the same day.

**Table 5:** Association of personality facets to previous day mental health symptoms and vice versa

	MHS -> Vulnerability		MHS -> Sociability		MHS -> Nonantagonism	
	***β***	***t***	***β***	***t***	***Β***	***t***
Step 1:						
MHS same day	0.52	40.87^***^	−0.38	−17.23^***^	−0.24	−14.38^***^
Step 2:						
MHS same day	0.50	36.50^***^	−0.37	−15.83^***^	−0.24	−13.96^***^
MHS 1 day before	0.04	2.78^**^	−0.01	−0.06	0.02	1.29
Step 3:						
MHS same day	0.50	36.42^***^	−0.37	−15.81^***^	−0.25	−14.08^***^
MHS 1 day before	0.04	2.70^**^	−0.02	−0.07	0.01	0.64
MHS 2 days before	−0.003	−0.27	0.01	0.49	0.03	1.98
	**Vulnerability -> MHS**		**Sociability -> MHS**		**Nonantagonism -> MHS**	
	***β***	***t***	***β***	***t***	***Β***	***T***
Step 1:						
Pers. same day	0.59	40.94^***^	−0.31	−22.41^***^	−0.33	−17.30^***^
Step 2:						
Pers. same day	0.58	38.31^***^	−0.30	−21.16^***^	−0.32	−16.54^***^
Pers. 1 day before	0.05	3.24^**^	−0.03	−2.41^*^	−0.03	−1.41
Step 3:						
Pers same day	0.58	29.34^***^	−0.30	−21.07^***^	−0.33	−16.53^***^
Pers. 1 day before	0.05	3.42^***^	−0.03	−2.31^*^	−0.03	−1.62
Pers. 2 days before	−0.02	−1.12	−0.01	−0.16	0.03	1.28

*Pers* Personality, *MHS* Mental Health Symptoms.

*p_FDR_ < 0.05, **p_FDR_ < 0.01, ***p_FDR_ < 0.001.

## Discussion

The present 75-day intensive longitudinal study evaluated menstrual cycle-related changes in personality facets and explored whether these changes were related to changes in mental health symptoms and libido. As hypothesised, we observed increased stress vulnerability (a facet of neuroticism) but reduced sociability (a facet of extraversion) and non-antagonistic orientation (a facet of agreeableness) during the peri-menstrual phase compared to the peri-ovulatory phase, but no menstrual cycle-related changes in other personality facets. Changes in personality facet expressions were, as expected, related to increased mental health symptoms during the premenstrual phase, with weaker associations to increased libido during the peri-ovulatory phase. Importantly, there was strong evidence for highly individualised trajectories of personality expressions, mental health symptoms, and libido along the menstrual cycle. Whereas some participants showed strong changes aligning with the average group trajectory, others showed no changes or trajectories opposite the group average. Exploratory moderation analyses suggested stronger increases in stress vulnerability in participants with stronger mental health symptoms in the premenstrual phase, though changes in sociability and non-antagonistic orientation were not significantly moderated by premenstrual mental health symptoms. Analyses of time-lagged associations suggested that changes in stress vulnerability both followed and preceded changes in mental health symptoms, while changes in sociability preceded but did not follow changes in mental health symptoms. Non-antagonistic orientation was only associated to same day mental health symptoms. Associations of personality facets to ovarian hormones were limited (see Supplementary Tables 1 and 3), though mental health symptoms were negatively, and libido positively, associated with estradiol levels (mirroring the cycle phase findings in Fig. 1).

One possible explanation for the peri-ovulatory increase in sociability, as well as the peri-menstrual increase in stress vulnerability and decrease in non-antagonistic orientation may lie in the ovarian hormone modulation of major neurotransmitter systems^46^. For example, estradiol increases dopaminergic signalling, which has been linked to positive emotionality^47^, a key concept related to sociability^48^. Different baseline levels in these neurotransmitter systems may also explain the highly individualised trajectories of personality facets along the menstrual cycle observed in the present study. Our findings are consistent with proposals suggesting that the mid-to-late luteal phase is a window of vulnerability for stress-related disorders^10,19^, while simultaneously highlighting that this may only be the case for some women. Unlike previous studies, the intensive longitudinal design allowed us to explore individual trajectories as well as time-lagged associations between stress vulnerability and mental health symptoms.

The strong variability in menstrual cycle trajectories of all variables is in line with previous literature highlighting inter-individual differences in premenstrual (mental health) symptom strength^24,25^, and it may explain inconsistencies among findings relating to positive and negative affect changes along the menstrual cycle^8–11^. Importantly, inter-individual differences in menstrual cycle-related trajectories of all variables, except hormone levels, were much stronger than associations to menstrual cycle phases. This is consistent with previous findings demonstrating similar affect variability across women with different hormonal milieus and men^49^. For positive and negative affect specifically, our study highlighted no changes in the whole sample but strong moderation effects by premenstrual mental health symptoms strength. However, not all menstrual cycle-related changes were moderated by premenstrual mental health symptoms strength, suggesting individual variability in menstrual cycle trajectories beyond PMS symptomatology.

Time-lagged associations allowed us to shed some light into a much-debated question in resilience research, i.e., whether stress vulnerability precedes or follows mental health problems. Our results provide support for associations in both directions, suggesting that stress vulnerability and mental health symptoms exacerbate each other. This finding is highly relevant to resilience research even outside the context of menstrual cycle research. However, the fact that both variables also shift cyclically along the menstrual cycle may suggest that a reset occurs during the follicular phase, with estradiol-driven neuroplasticity providing one potential mechanism for this reset. The fact that personality facets differed most prominently between the high estradiol peri-ovulatory and peri-menstrual phase, with intermediate findings during other cycle phases, provides partial support for this idea and is in line with the view of the peri-ovulatory phase as a window of resilience against stress-related disorders^5^.

Interestingly, the bidirectional association between personality shifts and mental health symptoms was only confirmed for stress vulnerability and not for other personality facets. Importantly, reductions in sociability preceded increases in mental health symptoms, but changes in mental health symptoms did not elicit changes in sociability. This finding is in line with a large literature highlighting the importance of social participation for mental health at the between-person level^50^. However, to the best of our knowledge, our study is the first to demonstrate a similar association at the intra-individual level, though sociability does not measure social participation per se, but individuals’ tendency and willingness to engage in social contact. Given that changes in sociability were a precedent rather than a consequence of changes in mental health symptoms, the mechanism underlying changes in sociability remains to be explored. In the present data set, changes in sociability were neither preceded by changes in physical symptoms nor by changes in libido. However, sociability was strongly related to mental health symptoms, physical symptoms, and libido on the same day. It is possible that participants’ sociability responds to their mental and physical health on the same day but then shows persisting associations to mental health the next day.

Premenstrual reductions in sociability were paralleled by premenstrual reductions in non-antagonistic orientation, which did not show time-lagged associations to mental health but only related to mental health symptoms on the same day. Given that irritability is one of the mental health symptoms associated with PMDD, it is possible that changes in irritability were also reflected in participants’ ratings of their non-antagonistic orientation. However, unlike for sociability, this did not show persisting associations to mental health the next day.

Finally, the conscientiousness-related facets dependability and achievement orientation did not show changes along the menstrual cycle, suggesting that—as with other hormone links to behaviour—hormone links to personality facets are specific to certain facets, but not others. Intra-individual variability in conscientiousness-related facets appears not to be hormonally mediated. This is consistent with findings that conscientiousness appears to change substantially during adolescence^36^ but tends to show stronger rank-order stability than other Big-five personality traits during adulthood. Thus, personality facets differ in their sensitivity to various stimuli.

While the intensive longitudinal design allows a fine-grained day-to-day analysis of associations between hormones, personality facets, and mental health symptoms—which is the biggest strength of this study—it comes with a few trade-offs that limit the generalizability of our results. Notably, only a selection of personality facets could be investigated by relatively short scales. Although these scales were suitable for intra-individual assessment, they were still derived from a trait questionnaire and not specifically developed for assessment of personality states. Thus, personality may not have been captured in its full complexity in the current study. For instance, openness to experiences could not be captured due to lack of suitable instruments. Furthermore, daily hormone assessments required a non-invasive approach via saliva sampling. Given that salivary assessments of ovarian hormones are less reliable than blood-based measures, associations to sex hormones as presented in the supplementary material should thus be interpreted with caution, as should associations with *libido*, given the reduced sample size for this variable. Finally, the study included mostly healthy participants with only low prevalence of premenstrual mental health symptoms. Given that the heightened premenstrual stress vulnerability appears to be particularly relevant for women with PMDD, future studies should consider focusing intensive longitudinal assessments of personality on these patients to gain a comprehensive picture of the relevance of menstrual cycle-related personality expressions for reproductive mood disorders.

In summary, the current results suggest that several personality facets remain relatively stable along the menstrual cycle, though state manifestations in stress vulnerability, sociability, and non-antagonistic orientation showed associations to the menstrual cycle. Nevertheless, the menstrual cycle-related shifts in these personality facets were relatively subtle and did not fully account for menstrual cycle-related changes in women’s mental health. Given that the current investigation was only able to include a selection of personality facets, it is possible that personality facet shifts related to women’s affective experiences along the menstrual cycle are more complex (compare also Giudice et al.^51^) and extend to other personality aspects beyond stress vulnerability, sociability, and non-antagonistic orientation. However, time-lagged analyses revealed important associations between personality facets and mental health beyond their association to the menstrual cycle, highlighting that intensive longitudinal designs can begin to provide answers to long-standing questions in personality research, such as whether mental health problems arise from certain personality traits or vice versa.

## Methods

### Participants

For this study, we recruited healthy female participants, aged 18–35 years, who reported no current diagnosis or history of psychiatric, neurological or endocrinological disorders or medication use, and had not used hormonal contraceptives in the past 6 months. Given the low base rate of PMDD diagnoses^26^, a diagnosis of PMDD was not an exclusion criterion. The most important inclusion criterion was a regular menstrual cycle of 21–35 days in length and a variation between individual cycles of less than 7 days. Before entering the study, participants provided data of at least three menstrual cycles from either a menstrual cycle app or calendar. The sample overlaps to 57% with the sample reported in Pletzer et al. (2024)^3^.

Ninety-five participants started the study, but 25 dropped out before completing their first cycle or missed more than 5 consecutive days between diary entries, and thus, were not included in the analyses. In addition, data from one participant had to be excluded due to pregnancy and one due to anovulatory cycles. In total, data from 68 participants (mean age: *M* = 25.29, *SD* = 4.76 years) were included in the analyses. All participants identified as women and were White/Caucasian. Education level was high, with 91% having passed general qualification for university entrance. Average cycle length was 28.16 days (*SD* = 2.62 days), with an average variation between cycles of 2.23 days (*SD* = 1.80 days). Twelve percent of participants had previously given birth, and 76% of participants had previously used hormonal contraception. Diagnostic criteria of the Daily Rating of Severity of Problems (DRSP) suggest a PMDD diagnosis in 3% of the participants and PMS for an additional 12%.

### Ethics

Participants provided informed written consent to participate in the study. All methods conformed to the Declaration of Helsinki and were approved by the University of Salzburg’s ethics committee (GZ 28/2018).

### Procedures

Participants were recruited via university newsletters, social media, and word of mouth and represent a convenience sample. Before the first online diary survey/entry, participants took part in a screening session, during which we obtained demographic information and medical history relating to the inclusion and exclusion criteria, an estimate of general cognitive ability based on the screening version of the Advanced Progressive Matrices (APM^52^), as well as a self-report-based estimate of premenstrual symptom strength using the Premenstrual Symptom Screening Tool (PSST^53^). The screening session was also used to explain the procedures related to ovulation test kits.

Following the screening session, participants completed a 30 min online diary every day between 5 p.m. and 10 p.m. for 75 days. The timeframe was chosen to ensure comparability between days by accounting for potential circadian fluctuations in steroid levels and mood. Participants provided two saliva samples per day, one before they started the diary, and one after completing the diary, which were frozen at −20 °C in their home freezer until picked up by our lab technician. Participants completed ovulation tests starting 5 days before the expected ovulation and recorded positive ovulation tests (Pregnafix®) as well as menses onsets in the online diary. Saliva tubes and ovulation tests were delivered to their home prior to the study. Hormone levels were analysed after pooling the two samples for each test day (for details, see the Hormone analysis section below). Participants received a small compensation for their time and effort of up to EUR 150, depending on the percentage of daily diaries completed.

### Daily diary questionnaires

Diaries consisted of: (i) a screening questionnaire assessing daily behaviour relevant to hormone measurements, including sleep, sport, stress, gender role, as well as caffeine, alcohol, nicotine, medication, and drug use, (ii) a personality assessment using an adaptation of the German Neuroticism Extraversion Openness-Five Factor Inventory (NEO-FFI^54^), (iii) an assessment of impulsivity using an adaptation of the non-planning subscale of the German Barratt Impulsiveness Scale^55^, (iv) a mood assessment using an adaptation of the German Positive and Negative Affect Schedule (PANAS^56^), (v) an assessment of mental health and physical symptoms typically associated with the premenstrual phase using the German Daily Rating of Severity of Problems (DRSP^57^), as well as (vi) an assessment of libido using an adaptation of the desire subscale of the Female Sexual Functioning Index (FSFI^58^). A subsample of participants additionally completed daily cognitive assessments, described elsewhere^3^.

#### Personality assessment

The NEO-FFI scale is a 60-item self-report instrument originally developed by Costa and McCrae (1994)^59^, which includes 5 personality trait subscales with 12 items for each of the *Big Five* factors Neuroticism (N), Extraversion (E), Openness to Experience (O), Agreeableness (A), and Conscientiousness (C). For the current study, we adapted the German version^54^ with kind permission of Hogrefe Publishing. Instructions of the original measure were modified to comply with state rather than trait assessment, asking participants to select the answer that best described them on that day. The answering format was kept as a five-point Likert scale ranging from *strongly disagree* (1) to *disagree* (2), *neutral* (3), *agree* (4), or *strongly agree* (5). Relevant for the current study are those NEO items that have been shown to represent facets that can be validly assessed within and between individuals at the same time^42^. Those include the neuroticism facet stress vulnerability (NEO items 11, 41, 51), the extraversion facet sociability (NEO items 2, 17, 27), the agreeableness facet non-antagonistic orientation (NEO items 14, 24, 39) as well as the conscientiousness facets achievement orientation (NEO items 25, 35, 60) and dependability (NEO items 20, 40, 50). Each facet score was obtained by averaging the ratings of the three individual items. Cronbach’s alphas ranged from 0.69 to 0.85 in the current sample.

#### Positive and Negative Affect Schedule (PANAS)

The PANAS^56^ is a validated 20-item instrument including 10 positive and 10 negative affect adjectives. Participants rated how much each adjective described their mood on that day on a 5-point Likert scale (1 = *not at all*, 5 = *extremely*). Scores for positive and negative affect were obtained by averaging the ratings of the respective 10 items. Cronbach’s alpha was 0.91 for positive affect and 0.85 for negative affect in the current sample.

#### Daily Rating of Severity of Problems (DRSP)

With the DRSP^57^, participants track common symptoms of premenstrual dysphoric disorder (PMDD) according to the DSM-V, as well as their impact on 3 areas of daily life on a 6-point Likert scale (1 = *not at all*, 6 = *extremely*). The symptom detail can be adjusted to gain different levels of information about a specific symptom area. In the current study, we included the general formulation of nine psychological (core symptoms: depression, anxiety, mood lability, irritability; associated symptoms: fatigue, loss of interest, feeling overwhelmed, problems concentrating, appetite changes) and the detailed formulation of five physical symptoms (headaches, nausea, breast swelling or pain, weight gain, joint pain). Daily scores were calculated separately for mental health and physical symptoms by averaging the scores of the respective symptom items. Cronbach’s alpha was 0.87 for mental health symptoms and 0.60 for physical symptoms in the current sample. In addition, the DRSP was used to assess the suitability of a potential PMS/PMDD diagnosis. Following Eisenlohr-Muhl et al.^60^, we considered a diagnosis of PMDD if at least five symptoms (at least one being a core psychological symptom) showed a change of more than 30% between cycle days 6–9 and the 5 days before the next menstruation and were accompanied by substantial impact on daily life over two consecutive cycles. We considered PMS if at least three symptoms fulfilled these criteria, as implemented in the PSST^53^.

#### Female Sexual Functioning Index (FSFI)

The *desire* subscale of the FSFI^58^ consists of two items assessing the frequency and level of sexual desire/interest, respectively, on a five-point Likert scale. For the present study, instructions were adapted to assess sexual desire over the past 24 h. Please note that only a subsample of 39 participants completed this scale daily, given that it was a late addition to the online diary due to sensitivity considerations.

### Hormone analysis

To remove solid particles, saliva samples were centrifuged twice for 15 and 10 min, respectively, at 3000 rpm. To obtain an average hormone level across the diary session, taking into account the pulsatile release pattern of ovarian hormones^61^, the two saliva samples from the same day were pooled. Estradiol and progesterone levels were assessed in duplicate from the pooled samples using Salimetrics salivary ELISA kits (www.salimetrics.com). The sensitivities according to the manufacturer’s specifications were 0.1 pg/ml for estradiol and 5 pg/ml for progesterone. Samples of the same participant were assayed on the same ELISA plate, and hormone analysis was repeated for participants with coefficients of variance (CoV) of more than 25% between duplicates of the same sample. On average, intra-assay CoV was 6.9% for estradiol and 9.7% for progesterone, while inter-assay CoV was 8.2% for estradiol and 15.8% for progesterone.

### Statistical analysis

Statistical analysis was carried out in R 4.2.3. On average, participants completed 73.75 (*SD* = 8.22) diaries, and only 169 of 5981 data points (˂3%) were missing. To account for measurement noise in menstrual cycle comparisons, personality facet scores, mood, and libido scores were smoothed using a moving average over 5 consecutive days (compare Guevarra et al.^10^). Cycle phases were defined based on backwards counting of cycle days from the onset of menses (compare Schmalenberger et al.^62^): (i) cycle days −3 to +2 encompassed the peri-menstrual phase, (ii) cycle days −9 to −5 encompassed the mid-luteal phase characterised by elevated progesterone levels, (iii) cycle days −15 to −12 encompassed the late follicular and ovulatory phase characterised by elevated estradiol levels, and (iv) cycle days prior to day −20, but after day +3 encompassed the early follicular phase including menses. All cycle days were included in the statistical analyses to obtain sufficient statistical power for the assessment of intra-individual variability in menstrual cycle-related trajectories using random slope analyses.

Given the multi-level structure of the data and the goal to model inter-individual variability in intra-individual changes, menstrual cycle-dependent changes in hormone levels, personality, affect, mental health symptoms, and libido were each evaluated in the context of a linear mixed effects model using the *lme* function of the *lme4* package^63^ including menstrual cycle phase (dummy-coded) as a fixed effect, as well as random intercepts and random slopes across participants. In addition, measurement day was included as a fixed effect (continuous, first session set to 0 as a reference point) to control for habituation or practice effects. Accordingly, across all dependent variables, the following measurement model was applied: Dependent Variable ~ CyclePhase + day + (1 + CyclePhase|PNr), where *1* denotes the intercept and *PNr* the participant number. To evaluate the extent of inter-individual variability, the random slopes model was compared against a random intercept model using the *anova* function. Since multiple dependent variables were addressed, *p*-values were FDR-corrected for multiple comparisons, and frequentist statistics were accompanied by Bayesian analyses using the *lmBF* function of the *BayesFactor* package^64^. Bayes factors provide information on the relative likelihood (via odds ratios) of two statistical models, given the data: a null model (here, excluding cycle phase) and an alternative model (here, including cycle phase). For instance, a Bayes factor of three supporting the alternative model indicates that the model including *cycle phase* is three times more likely than the model excluding cycle phase, i.e., the null model. The prior distribution was a zero-centred Cauchy distribution with a scale of 0.707.

To explore the association between menstrual cycle-related changes in personality facets and changes in mental health symptoms, affect, and libido, we employed a hierarchical approach. First, we evaluated zero-order correlations between individually centred variables to address associations between intra-individual fluctuations. Second, we added (i) mental health symptoms (Personality ~ CyclePhase + MentalHealth + day + (1 + CyclePhase|PNr)) and (ii) libido (Personality ~ CyclePhase + MentalHealth + Libido + day + (1 + CyclePhase|PNr)) as additional covariates in the models assessing menstrual cycle-related changes in personality. Both these analyses are designed to reveal time-locked associations, i.e., associations between personality and mental health on the same day. Third, the time-lagged value of each predictor from the day before was added as an additional predictor to these models (Personality ~ CyclePhase + MentalHealth + MentalHealth_t-1_ + day + (1 + CyclePhase|PNr), to begin to reveal the directionality of associations. Please note that for these latter analyses, we did not employ the smoothed variables since we were interested in day-to-day variations rather than averages across whole cycle phases.

## Supplementary information

Supplementary information

## Data Availability

Data is available at https://osf.io/46d92/.

## Code availability

Code pertaining to the analysis is available at https://osf.io/46d92/.
