# Supplementary material for: Menstrual cycle variations in stress vulnerability and sociability relate to mental health symptoms and libido
Source: NPJ Womens Health. 2026 Apr 17;4(1):18. doi: 10.1038/s44294-026-00140-z (PMC13090119; doi:10.1038/s44294-026-00140-z)
Supplement: Supplementary file 1 — Supplementary information [file 44294_2026_140_MOESM1_ESM.pdf]

Supplementary material

Menstrual cycle variations in stress vulnerability and sociability relate to mental health symptoms and libido.

A. Associations of personality facets, affect and libido to salivary estradiol and progesterone levels

Hormonal associations using smoothed variables are summarized in **Supplementary Table 1**. Stress vulnerability, as well as positive and negative affect showed no associations to sex hormones. Higher sociability was associated with higher estradiol and lower progesterone levels. Likewise, higher non-antagonistic orientation was associated to lower progesterone levels. The conscientiousness facet dependability was not associated to sex hormone levels, though interestingly, achievement orientation was negatively related to estradiol. Higher estradiol levels were also related to lower premenstrual symptoms and higher libido.

	**Estradiol**			**Progesterone**			**Estradiol*Progesterone**		
	**β**	**t**	**BF_{10}**	**β**	**t**	**BF_{10}**	**β**	**t**	**BF_{10}**
**Stress Vulnerability**	-0.02	-1.24	0.19±1.09%	<0.01	0.28	0.10±1.37%	0.02	2.23	1.05±1.85%
**Sociability**	**0.07**	**3.82*****	**184.34±0.79%**	**-0.05**	**-3.79*****	**85.35±0.92%**	-0.02	0.59	0.11±0.55%
**Nonantagonistic O.**	0.01	0.80	0.13±1.07%	**-0.05**	**-5.12*****	**$1*10^5$±0.85%**	-0.01	-0.65	0.10±1.90%
**Achievement O.**	**-0.05**	**-3.10****	**14.10±0.92%**	-0.03	-2.35	1.86±0.85%	-0.01	-0.60	0.11±0.46%
**Dependability**	-0.03	-1.70	0.38±0.81%	-0.02	-1.32	0.15±0.72%	0.02	1.62	0.37±0.81%
**Mental Health S.**	**-0.05**	**-3.48****	**124.22±4.09%**	<0.01	-0.16	0.08±4.04%	0.01	1.27	0.18±6.84%
**Physical S.**	**-0.06**	**-3.45****	**70.15±4.11%**	-0.01	-1.04	0.25±4.03%	-0.02	-2.04	0.67±6.66%
**Positive Affect**	-0.03	-2.43	1.36±0.51%	-0.01	-1.06	0.10±0.55%	0.02	2.46	1.95±0.93%
**Negative Affect**	-0.01	-0.49	0.12±0.98%	0.01	1.02	0.16±0.88%	<0.01	0.38	0.11±0.94%
**Libido**	**0.17**	**6.61*****	**$1*10^9$±0.77%**	**-0.10**	**-5.38*****	**$4*10^4$±3.54%**	0.03	1.67	0.44±1.13%

Supplementary Table 1: Associations of personality, mental health and libido with estradiol, progesterone and their interaction. O. = Orientation, S. = Symptoms, *$p_{FDR} < 0.05$, **$p_{FDR} < 0.01$, ***$p_{FDR} < 0.001$. BF_{10} = Bayes factor in support of the alternative hypothesis.

B. Moderation of menstrual cycle shifts in personality facets

Interactions between menstrual cycle phase and premenstrual symptom severity are displayed in **Supplementary Table 2**. Significant moderation by premenstrual symptom severity was observed for *stress vulnerability*, as well as positive and negative affect, indicating stronger changes in stress vulnerability and affect in women with higher premenstrual symptom severity. No significant interactions were observed for other personality facets or libido. Age and prior contraceptive use did not significantly moderate menstrual cycle related shifts in personality facets, affect or libido.

	**Cycle Phase*PMS**		**Cycle Phase*Age**		**Cycle Phase*Prior HC**	
	**η_p^2**	**F**	**η_p^2**	**F**	**η_p^2**	**F**
**Vulnerability**	0.01	4.35^{**}	<0.01	0.94	<0.01	1.74
**Sociability**	<0.01	3.44	<0.01	3.18	0.01	$3.94^{\sim}$
**Nonantagonistic O.**	<0.01	1.06	<0.01	3.06	<0.01	0.65
**Achievement O.**	<0.01	0.88	<0.01	1.26	<0.01	0.59
**Dependability**	<0.01	1.15	<0.01	2.09	<0.01	2.09
**Positive Affect**	0.01	7.39^{***}	<0.01	1.16	<0.01	0.17
**Negative Affect**	0.01	11.28^{***}	<0.01	2.92	<0.01	0.52
**Libido**	<0.01	0.17	<0.01	1.65	<0.01	0.52

Supplementary Table 2: Moderation of association to cycle phase by premenstrual symptom severity (PMS), age and prior use of hormonal contraceptives (HC)

C. Time-lagged associations of personality facets to ovarian hormone levels

Without smoothing, none of the personality facet scores were associated to estradiol either on the same day or one or two days before. Thus, smoothing, as was performed in supplementary A, appears to be particularly relevant to detect hormone-behaviour associations over a sliding window of five days, but associations cannot be time-locked to specific days.

Sociability and non-antagonistic orientation were negatively associated to progesterone on the same day, but not to progesterone one or two days before.

	**Stress Vulnerability**		**Sociability**		**Nonantagonistic O.**	
	**β**	**t**	**β**	**t**	**β**	**t**
**Step 1:**						
E same day	0.01	1.06	<0.01	-0.01	-0.01	-0.67
**Step 2:**						
E same day	0.02	1.08	<0.01	0.07	-0.01	-0.55
E 1 Day before	<0.01	-0.20	-0.01	-0.38	-0.01	-0.57
**Step 3:**						
E same day	0.02	1.09	<0.01	0.03	-0.01	-0.59
E 1 day before	<0.01	-0.20	-0.01	-0.49	-0.01	-0.70
E 2 days before	<0.01	-0.20	0.01	0.63	0.01	0.77
	**Stress Vulnerability**		**Sociability**		**Nonantagonistic O.**	
	**β**	**t**	**β**	**t**	**β**	**t**
**Step 1:**						
P same day	0.01	0.47	-0.04	-2.43*	-0.02	-1.97*
**Step 2:**						
P same day	0.01	0.91	-0.03	-1.32	-0.02	-1.44
P 1 Day before	-0.01	-0.92	-0.02	-1.19	<0.01	-0.32
**Step 3:**						
P same day	0.01	0.81	-0.02	-1.25	-0.02	-1.50
P 1 day before	-0.02	-0.96	-0.02	-1.02	-0.01	-0.49
P 2 days before	0.01	0.31	<0.01	-0.17	0.01	0.46

Supplementary Table 3: Association of personality facets to previous day hormone levels. O. = Orientation, E = Estradiol, P = Progesterone, *$p_{FDR} < 0.05$, **$p_{FDR} < 0.01$, ***$p_{FDR} < 0.001$.
